# Spatial patterns and socio-environmental determinants of gonorrhea incidence in China

**DOI:** 10.3389/fpubh.2025.1698923

**Published:** 2025-12-09

**Authors:** Ke Hu, Xingjin Yang, Yu Cai, Chaojie Li, Xing Zhang, Di Xiao, Mingyang Yu

**Affiliations:** 1 Xiamen Haicang Hospital, Xiamen, Fujian, China; 2 QianDongNanZhou Center for Disease Control and Prevention, Guizhou, China; 3 Shanghai Municipal Hospital of Traditional Chinese Medicine, Shanghai, China; 4 Xingtai Center for Disease Control and Prevention, Xingtai, Hebei, China; 5 Nanjing Lishui Dongping Street Health Center, Nanjing, Jiangsu, China; 6 Community Health Service Center of Jiuxian Tongliang District, Chongqing, China; 7 Fuwai Central China Cardiovascular Hospital, Zhengzhou, Henan, China

**Keywords:** gonorrhea incidence, spatial autocorrelation, multiple linear regression, spatial error model, Geodetector

## Abstract

**Introduction:**

Gonorrhea is a major sexually transmitted infection in China, showing distinct regional clustering and spatial heterogeneity. Understanding its geographical distribution and influencing factors is crucial for targeted prevention.

**Methods:**

We analyzed 2022 gonorrhea incidence across 31 Chinese provinces using both traditional and spatial statistical approaches, including descriptive statistics, spatial autocorrelation, multiple linear regression (MLR), and spatial error models (SEM). Factors from five categories—economic, demographic, environmental, educational, and healthcare-related—were examined. The Geodetector method was additionally used to assess factor contributions and interactions.

**Results:**

Three key findings emerged: (1) Significant regional disparities in gonorrhea incidence were observed, with high-high clusters detected in southern provinces and low-low clusters in northern regions.; (2) SEM outperformed MLR, confirming stronger effects of illiteracy rate, bed utilization rate, sex ratio, and PM_2.5_ concentration while demonstrating better model fit (higher R^2^, log-likelihood; lower AIC); (3) Sex ratio was identified as a core determinant, with interaction effects (particularly bed utilization rate and sex ratio) amplifying individual impacts.

**Conclusion:**

These results support spatially tailored intervention strategies that integrate sociodemographic and environmental factors for effective gonorrhea prevention.

## 1 Introduction

Gonorrhea, one of the most prevalent sexually transmitted infections in China, reported 96,313 cases in 2022, ranking as the fourth most common notifiable infectious disease nationwide, following viral hepatitis, tuberculosis, and syphilis (1). In China, gonorrhea incidence exhibits significant regional clustering and spatial heterogeneity. Studies have shown that disparities in gonorrhea incidence exist not only at the provincial level but also display aggregated distribution patterns in smaller administrative units (e.g., counties or districts). For instance, data from Zhejiang Province (2016–2020) revealed that gonorrhea incidence was significantly higher in northern and central regions (e.g., Hangzhou, Jiaxing) compared to the south. Spatial autocorrelation analysis (Moran’s I index) further confirmed its spatial clustering, with hotspot areas primarily concentrated in highly urbanized districts (2). This spatial heterogeneity may be closely linked to regional disparities in socio-environmental determinants of health (3, 4).

The epidemiology of gonorrhea is shaped by a complex interplay of multidimensional factors. Socioeconomically, lower per capita GDP is associated with higher incidence rates, likely due to reduced healthcare access in economically disadvantaged regions (5). Urbanization exerts a bidirectional influence, wherein expanded sexual networks may enhance transmission, whereas concentrated medical infrastructure could mitigate disease incidence (2). Demographic analyses reveal that areas with elevated illiteracy rates, indicative of poorer health literacy, demonstrate significantly higher gonorrhea prevalence (6–8), with additional influences from gender ratio imbalances (2, 9, 10). Healthcare system factors, particularly the density of physicians per 1,000 population and hospital bed utilization rates, critically determine case detection and treatment efficacy, where resource-limited areas often experience surveillance gaps leading to underreporting and treatment delays (11). Environmental determinants, including air pollution levels, have emerged as potential drivers of transmission dynamics (12, 13). The spatial heterogeneity of these interacting factors collectively determines the geographic distribution patterns of gonorrhea across regions.

Spatial regression models (e.g., Spatial Error Model and Spatial Lag Model) effectively address spatial autocorrelation issues neglected by conventional statistical methods. By constructing spatial weight matrices, these models can not only identify the direct effects of influencing factors on local areas but also quantify their spatial spillover effects (i.e., spatial dependence) on neighboring regions (14–16). In contrast, the Geodetector method, based on the principle of spatial stratified heterogeneity, demonstrates unique advantages: it can precisely quantify the independent contribution of each risk factor to spatial variations in disease incidence through its factor detector, while effectively identifying interaction effects between different factors (17, 18). Owing to its methodological strengths in analyzing disease spatial transmission mechanisms, Geodetector has been successfully implemented across multiple public health research domains, particularly in disease risk zoning, health resource allocation optimization, and environmental health risk assessment (19).

The spatial heterogeneity of gonorrhea incidence results from the nonlinear interactions of multiple socioeconomic factors, necessitating spatial analytical approaches to uncover underlying geographical patterns. The application of spatial regression models and Geodetector can overcome the limitations of conventional epidemiological analyses, thereby providing scientific evidence for identifying high-risk areas and optimizing resource allocation.

## 2 Methods

### 2.1 Data

Building upon the theoretical framework established in the background section and considering data availability, this study analyzed provincial-level data from 31 Chinese mainland administrative units (provinces, autonomous regions, and centrally administered municipalities) by integrating seven socio-environmental indicators in 2022 across five dimensions: economic development, population structure, environmental condition, education level, and healthcare resources (Table 1). Data were systematically compiled from authoritative sources: gonorrhea incidence rates from the most recent 2023 China Health Statistical Yearbook, PM_2.5_ concentrations from provincial environmental reports, and all remaining explanatory variables from the China Statistical Yearbook.

**Table 1:** Key factors selected for analysis categorized into economic development, education level, healthcare resources, population structure, and environmental condition.

Categories	Factors
Economic development	GDP per capita
	Urbanization rate
Education level	Illiteracy rate
Healthcare resources	Number of licensed physicians per 1,000 population
	Hospital bed utilization rate
Population structure	Sex ratio
Environment condition	PM_2.5_

### 2.2 Descriptive analyses

The spatial distribution characteristics of gonorrhea incidence in the 31 districts were illustrated through thematic mapping.

### 2.3 Spatial autocorrelation analysis

The methodological framework for spatial autocorrelation analysis primarily comprises the Global Moran’s I and Local Moran’s I indices.

#### 2.3.1 Global Moran’s I

As a fundamental tool in spatial econometrics, the Global Moran’s I index is principally employed to measure spatial dependence at the aggregate regional level. Its mathematical formulation is expressed as Equation 1 (20):


$$I=\frac{n\sum_{i=1}^{n}\sum_{j=1}^{n}W_{\mathrm{ij}}(x_{i}-\bar{x})(x_{j}-\bar{x})}{\sum_{i=1}^{n}\sum_{j=1}^{n}W_{\mathrm{ij}}\sum_{i=1}^{n}{\left(x_{i}-\bar{x}\right)}^{2}}$$ (1)

The Global Moran’s I index, ranging from −1 to 1, quantifies spatial dependence through the formula incorporating sample size (*n*), spatial weight matrix elements (*W*_ij_), observed values (*x*_i_, *x*_j_), and their mean ( $\bar{x}$ ). Values demonstrate distinct spatial patterns: significant positive autocorrelation (0 < *I* ≤ 1) reflects clustering of similar values, negative autocorrelation (−1 ≤ *I* < 0) indicates dispersion of dissimilar values, while *I* ≈ 0 suggests random distribution. Statistical significance is assessed via standardized Z-scores (21).

#### 2.3.2 Local Moran’s I (LISA)

LISA primarily serves to uncover localized spatial heterogeneity patterns within study regions. By quantifying local spatial autocorrelation, it effectively identifies statistically significant spatial clusters. The computational formula is expressed as Equation 2 (22):


$$I_{i}=\frac{n(x_{i}-\bar{x})\sum_{j=1}^{n}w_{\mathrm{ij}}(n_{j}-\bar{n})}{\sum_{j=1}^{n}{\left(x_{j}-\bar{x}\right)}^{2}}$$ (2)

LISA adopts the same variable definition framework as the Global Moran’s I index to identify four distinct spatial association patterns: high-high clustering (HH-type) representing agglomeration of high-value areas, low-low clustering (LL-type) indicating concentration of low-value regions, high-low outliers (HL-type) showing isolated high values surrounded by low values, and low-high outliers (LH-type) featuring isolated low values encircled by high values. These spatial patterns, when validated by significance testing and visualized through LISA cluster maps, effectively characterize the spatial heterogeneity of the study region (23).

### 2.4 Multiple Linear Regression (MLR)

MLR is a multivariate statistical analysis method that quantifies the collective influence of multiple explanatory variables on a single response variable through the construction of a linear mathematical model (24). The standard formulation is expressed as Equation 3:


$$Y=\beta_{0}+\beta_{1}X_{1}+\beta_{2}X_{2}+\ldots +\beta_{p}X_{p}+\varepsilon$$ (3)

where *Y* represents the dependent variable, *X*_1_, *X*_2_, …, *X*_p_ denote the independent variables, *β*_0_ is the intercept term, *β*₁, *β*₂, …, *β*_p_ are the regression coefficients, and *ϵ* stands for the random error term following a normal distribution with mean zero.

The goodness-of-fit of mode was comprehensively evaluated using multiple metrics, including the coefficient of determination (R^2^), Akaike Information Criterion (AIC), and log-likelihood values. Additionally, to assess potential multicollinearity among the independent variables, diagnostic analysis was performed using variance inflation factors (VIF). Variables with VIF > 5 may exhibit multicollinearity (25).

### 2.5 Spatial regression models

#### 2.5.1 Spatial error model (SEM)

SEM is a specialized spatial econometric approach designed to address spatial autocorrelation in regression residuals. The model structure is specified as Equations 4, 5:


$$Y=X_{\beta }+u$$ (4)

$$u=\lambda W_{u}+\varepsilon$$ (5)

where *λ* denotes the spatial error coefficient and *W*_u_ represents the spatially lagged error term. By incorporating a spatial autocorrelation structure into the error terms, SEM effectively corrects the parameter estimation bias caused by residual spatial dependence in conventional regression analysis (26).

#### 2.5.2 Spatial lag model (SLM)

SLM effectively captures spatial dependence among study units by incorporating a spatial lag term of the dependent variable. The model is expressed as Equation 6 (27):


$$Y=\rho W_{Y}+X_{\beta }+\varepsilon$$ (6)

where *ρ* represents the spatial autoregressive coefficient, *W* denotes the spatial weight matrix, and *W*_Y_ indicates the spatially lagged dependent variable. This model is particularly suitable for quantifying spillover effects between spatial units.

### 2.6 Geodetector

The Geodetector is a statistical method based on the principle of spatial stratified heterogeneity, which systematically examines spatial differentiation patterns and their underlying drivers through four integrated analytical modules: the Factor Detector quantifying explanatory power using PD (Power of Determinant)-statistics, the Interaction Detector evaluating synergistic effects among multiple factors, the Risk Detector identifying high-risk spatial zones, and the Ecological Detector testing the significance of factor differences (28–30). This comprehensive framework enables rigorous investigation of complex spatial heterogeneity by simultaneously assessing individual factor contributions, interaction effects, risk distribution patterns, and statistical significance of spatial variations.

#### 2.6.1 Factor detector

The Factor Detector module within the Geodetector methodology is grounded in the theory of spatial stratified heterogeneity. It systematically evaluates the independent contribution of each explanatory variable to the spatial differentiation characteristics of the study target by constructing the PD-value (also called q statistic). The computational principle of this method lies in analyzing the difference between within-stratum variance and total variance, thereby achieving a precise measurement of the explanatory power of influencing factors (31). The model is expressed mathematically in Equation 7:


$$\mathrm{PD}=1-\frac{\sum_{h=1}^{L}N_{h}\sigma_{h}^{2}}{N\sigma^{2}}$$ (7)

Here, *N*_h_ denotes the sample size of the *h*-th stratum, σ_h_^2^ represents the variance of the *h*-th stratum, *N* is the total sample size, and σ^2^ is the total variance. The PD-value ranges between [0, 1], and its magnitude is positively correlated with the explanatory power of the variable. Specifically, the PD-value closer to 1 indicates a stronger influence of the factor on spatial differentiation.

#### 2.6.2 Risk detector

The Risk Detector is designed to assess the heterogeneity of spatial risk levels under stratified conditions of different influencing factors. Based on the statistical principle of independent samples t-test, this method identifies high-risk or low-risk areas in spatial distribution by testing the significance of mean differences across stratified sub-regions (28). See Equation 8 for the model formulation:


$$t_{{\bar{y}}_{h-1}-{\bar{y}}_{h-2}}=\frac{{\bar{Y}}_{h=1}-{\bar{Y}}_{h=2}}{{\left[\frac{\mathrm{Var}({\bar{Y}}_{h=1})}{n_{h=1}}+\frac{\mathrm{Var}({\bar{Y}}_{h=2})}{n_{h=2}}\right]}^{1/2}}$$ (8)

Where $\bar{Y}h$ represents the average gonorrhea incidence of layer *h*, *n*_h_ is samples, *Var* represents sample variance, *t* follows the Student’s-*t*-test distribution. The null hypothesis as follows (Equation 9):


$$H_{0}:{\bar{Y}}_{h=1}={\bar{Y}}_{h=2}$$ (9)

Rejection of the null hypothesis at the *α* level indicates statistically significant differences in the mean incidence rates across the two study regions.

#### 2.6.3 Ecological detector

The Ecological Detector is grounded in the Analysis of Variance (ANOVA) framework, employing the F-statistic to conduct significance testing on the explanatory power of influencing factors regarding spatial differentiation. Its core principle involves comparing within-stratum variance differences across factors, thereby quantitatively assessing whether each factor’s contribution to the formation of spatial patterns in geographical phenomena is statistically significant. The mathematical formulation of the model is given by Equations 10–12:


$$F=\frac{n_{X_{1}}(n_{x2}-1)\mathrm{SS}W_{X_{1}}}{n_{X_{2}}(n_{x1}-1)\mathrm{SS}W_{X_{2}}}$$ (10)

$$\mathrm{SS}W_{X_{1}}=\sum_{h=1}^{L_{1}}N_{h}\sigma_{h}^{2}$$ (11)

$$\mathrm{SS}W_{X_{2}}=\sum_{h=1}^{L_{2}}N_{h}\sigma_{h}^{2}$$ (12)

Where *n*_x1_ and *n*_x2_ represent the samples of two factors *x*_1_ and *x*_2_, respectively. *SSW*_x1_ and *SSW*_x2_ represent the sum of the within-strata variance of *x*_1_ and *x*_2_, respectively; *L*_1_ and *L*_2_ represent the number of layers of *x*_1_ and *x*_2_, respectively.

The null hypothesis (*H*_0_) of this test assumes that two influencing factors exhibit no significant difference in their explanatory power regarding the spatial differentiation of the dependent variable. By calculating the F-statistic and conducting hypothesis testing: if the observed *F*-value exceeds the critical value at a given significance level, the null hypothesis is rejected, indicating a statistically significant difference between the two factors in their ability to explain spatial differentiation.

#### 2.6.4 Interaction detector

The Interaction Detector is designed to investigate the synergistic effects of dual factors on geographical spatial differentiation. This method systematically compares the explanatory power (PD-value) of individual factors with the joint explanatory power (PD-value) of factor combinations, enabling precise identification of five characteristic interaction types (19): nonlinear-weakening, univariate-weakening, bivariate enhancement, independent, and nonlinear-enhancement effects (Table 2).

**Table 2:** Descriptions and classifications of interaction types between variables including weakened nonlinear, weakened univariate, enhanced bivariate, independent, and enhanced nonlinear interactions.

Description	Interaction
$\mathrm{PD}(X_{1}\cap X_{2})<\min(\mathrm{PD}(X_{1}),\mathrm{PD}(X_{2}))$	Weaken, nonlinear
$\min(\mathrm{PD}(X_{1}),\mathrm{PD}(X_{2}))<\mathrm{PD}(X_{1}\cap X_{2})<\max(\mathrm{PD}(X_{1}),\mathrm{PD}(X_{2}))$	Weaken, univariate
$\mathrm{PD}(X_{1}\cap X_{2})>\max(\mathrm{PD}(X_{1}),\mathrm{PD}(X_{2}))$	Enhanced, bivariate
$\mathrm{PD}(X_{1}\cap X_{2})=\mathrm{PD}(X_{1})+\mathrm{PD}(X_{2})$	Independent
$\mathrm{PD}(X_{1}\cap X_{2})>\mathrm{PD}(X_{1})+\mathrm{PD}(X_{2})$	Enhance, nonlinear

### 2.7 Classification methods for independent variables

Geodetector requires independent variables to be categorical, making the discretization of continuous variables a crucial preprocessing step. This study employs five mainstream discretization methods (32):

Natural breaks: Optimizes classification boundaries based on data distribution characteristics to maximize inter-category differences.
Quantile classification: Ensures balanced sample sizes across categories, suitable for skewed distributions.
Equal interval classification: Divides the value range into equal-width intervals, ideal for uniformly distributed data.
Geometric interval classification: Sets intervals based on geometric progression, tailored for exponentially distributed data.
Standard deviation classification: Partitions data based on intervals measured by n standard deviations from the mean, optimized for normal distributions.

The discretization optimization follows the principle of maximizing PD-value. By systematically comparing different classification methods with 3 to 8 intervals in factor detection, the optimal discretization scheme is determined. This standardized process significantly improves the model’s explanatory power.

### 2.8 Software

This study utilized ArcGIS 10.2 software (Esri)^1^ for the discretization of independent variables, spatial autocorrelation analysis, collinearity diagnosis, and map visualization. GeoDa 1.22 software^2^ was employed to construct multiple linear regression models and spatial regression models. All spatial analyses and the construction of spatial weight matrices were performed based on the Albers Equal Area Conic projection coordinate system to ensure measurement consistency and the reproducibility of results. The Geodetector analysis was conducted using its official Excel version.^3^ The foundational map data used in the study were sourced from the National Platform for Common Geospatial Information Services^4^ [approval number: GS (2024) 0650]. All statistical analyses adopted a two-tailed test, with the significance level set at *p* < 0.05.

## 3 Results

### 3.1 Spatial distribution characteristics and aggregation patterns of Gonorrhea incidence

**Figure 1:**
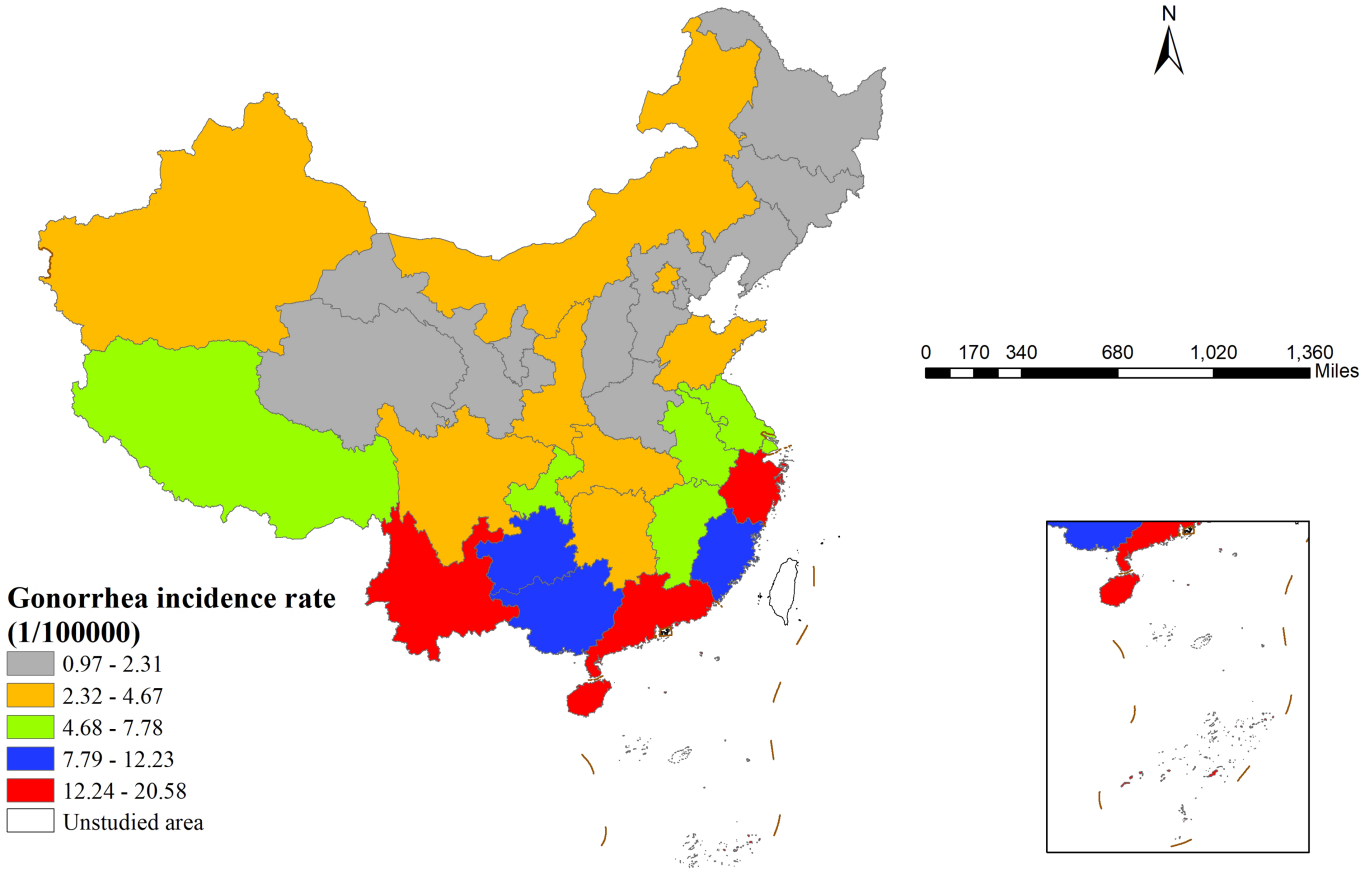
Spatial distribution of gonorrhea incidence rates across China in 2022 illustrating geographic heterogeneity and identifying regions with high and low incidence rates.

In 2022, gonorrhea incidence in China showed clear geographical variation, with southern regions generally experiencing higher rates than northern areas (Figure 1). Specifically, Zhejiang Province had the highest incidence rate (20.58 cases per 100,000 population), while Hebei Province had the lowest (0.94 cases per 100,000 population), indicating pronounced regional disparities.

**Figure 2:**
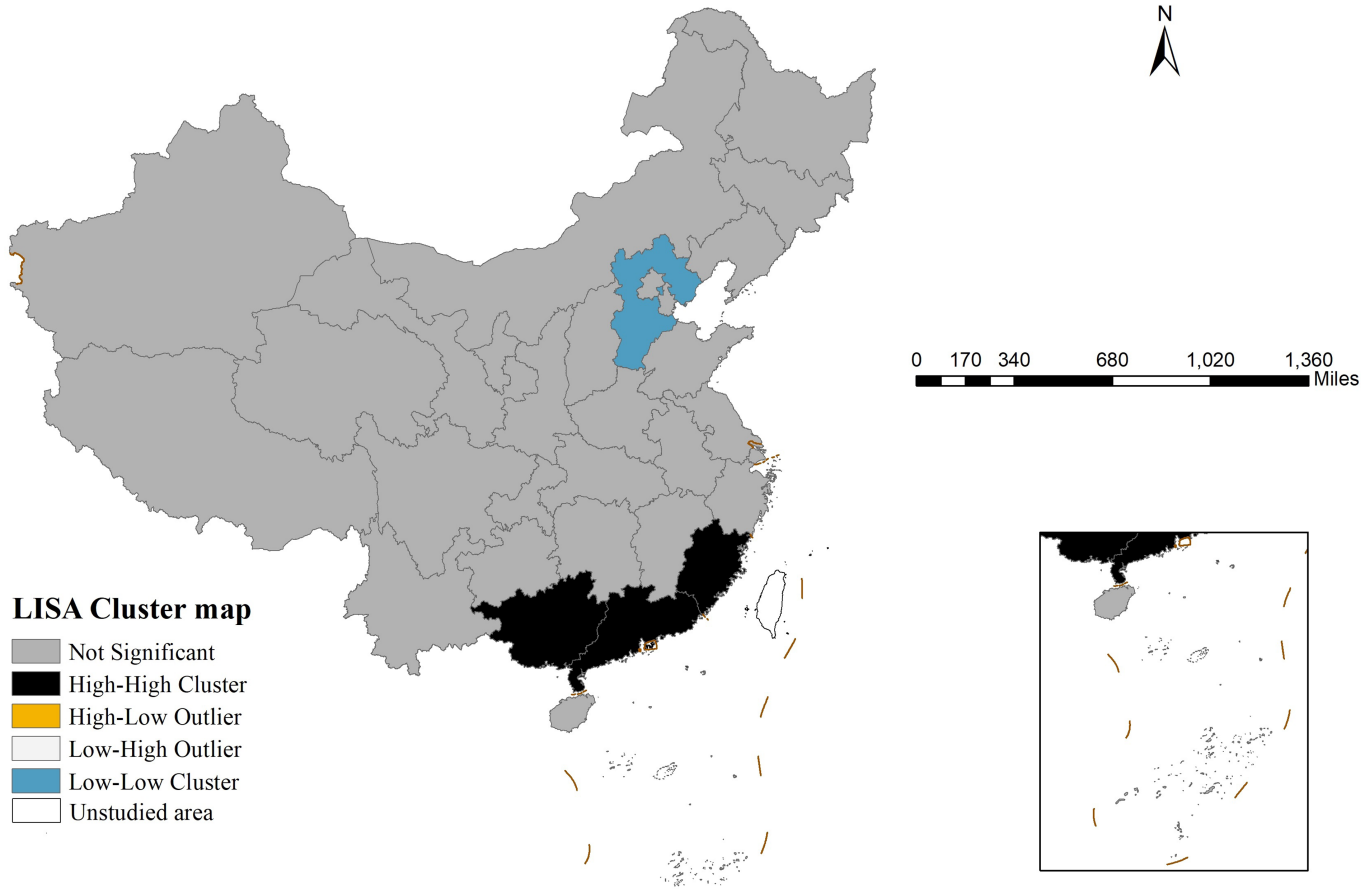
Local indicators of spatial association cluster map of gonorrhea incidence in China in 2022 revealing statistically significant spatial aggregation patterns including high-high clusters, low-low clusters, and spatial outliers.

Global spatial autocorrelation analysis revealed that the Moran’s I index for gonorrhea incidence in China in 2022 was 0.391 (*p* < 0.001), suggesting significant spatial clustering. LISA analysis results (Figure 2) showed two primary clustering patterns: high-high clusters were concentrated in southern coastal provinces, including Guangdong, Guangxi, and Fujian, whereas low-low clusters were predominantly located in northern provinces, with Hebei Province being the most typical example.

### 3.2 MLR results

Initial collinearity diagnostics revealed strong multicollinearity between GDP per capita and urbanization rate (VIF values of 7.2 and 7.92, respectively). After removing urbanization rate, all remaining variables had VIF values below 5. The preliminary multiple linear regression analysis (Table 3) indicated that illiteracy rate, bed utilization rate, sex ratio, and PM_2.5_ concentration had statistically significant effects on gonorrhea incidence. Additionally, Robust Lagrange Multiplier tests revealed statistically significant spatial dependence (error term: *p* = 0.010, lag term: *p* = 0.039), justifying the application of both SLM and SEM for further analysis.

**Table 3:** Comparison of model performance between the spatial error model and the multiple linear regression model presenting regression coefficients, statistical significance, r-squared, Akaike information criterion, and log-likelihood values for each predictor variable.

Factors	SEM		MLR	
	Coefficient	*p*-value	Coefficient	*p*-value
Intercept	−91.8309	0.0000	−90.9013	0.00169
GDP per capita	−0.0000	0.0879	−0.0000	0.2252
Illiteracy rate	−0.2483	0.00032	−0.2742	0.0278
Number of licensed physicians per 1,000 population	1.3817	0.2699	1.2368	0.4828
Hospital bed utilization rate	0.2690	0.00000	0.1764	0.01598
Sex ratio	0.8381	0.00000	0.8866	0.00039
PM_2.5_	−0.1901	0.00002	−0.2032	0.02986
R^2^	0.8086		0.7038	
AIC	157.287		164.498	
Log likelihood	−71.6434		−75.2489	

### 3.3 Spatial regression model results

The study employed SLM and SEM for spatial econometric analysis. Model testing results showed that the spatial lag model’s lag coefficient (*W*_Y_ = 0.054, *p* = 0.77801) did not reach statistical significance (*α* = 0.05). In contrast, the spatial error model’s error term coefficient was highly significant (*λ* = −0.982, *p* < 0.001). Based on these findings, the spatial error model was selected for further analysis.

Table 3 presents the estimation results of the spatial error model. Compared to MLR, this model not only confirmed the significant effects of illiteracy rate, bed utilization rate, sex ratio, and PM_2.5_ concentration on gonorrhea incidence (with stronger statistical significance for each variable) but also demonstrated superior model fit—evidenced by higher R^2^ and log-likelihood values, as well as a lower AIC value. These metrics collectively indicate that the spatial error model provides a better fit for the data.

### 3.4 Geodetector model results

#### 3.4.1 Discretization of independent variables

Five classification methods discretized seven variables into 3–8 categories. Factor detector calculated PD values for each scheme, and the optimal one (maximum PD value) was selected (Table 4).

**Table 4:** Results of the factor detection analysis showing the power of determinism values, statistical significance, classification method, and optimal class interval for each independent variable.

Factors	PD	*p*	Classification method	Classification interval
GDP per capita	0.384	0.230	Quantile	8
Urbanization rate	0.388	0.259	Natural breaks	8
Illiteracy rate	0.241	0.572	Quantile	8
Number of licensed physicians per 1,000 population	0.272	0.030	Quantile	3
Hospital bed utilization rate	0.504	0.048	Quantile	7
Sex ratio	0.619	0.017	Natural breaks	8
PM_2.5_	0.307	0.022	Geometric interval	3

#### 3.4.2 Factor detector results

Statistical results in Table 4 show that variables such as per capita GDP (*p* > 0.05), illiteracy rate (*p* > 0.05), and urbanization rate (*p* > 0.05) did not exhibit statistically significant associations with gonorrhea incidence. In contrast, other influencing factors demonstrated varying degrees of explanatory power (PD values ranging from 0.27 to 0.62). Among them, the sex ratio had the strongest explanatory power (PD = 0.62), while the number of physicians per 1,000 population showed relatively weaker explanatory power (PD = 0.27).

#### 3.4.3 Risk detector results

**Figure 3:**
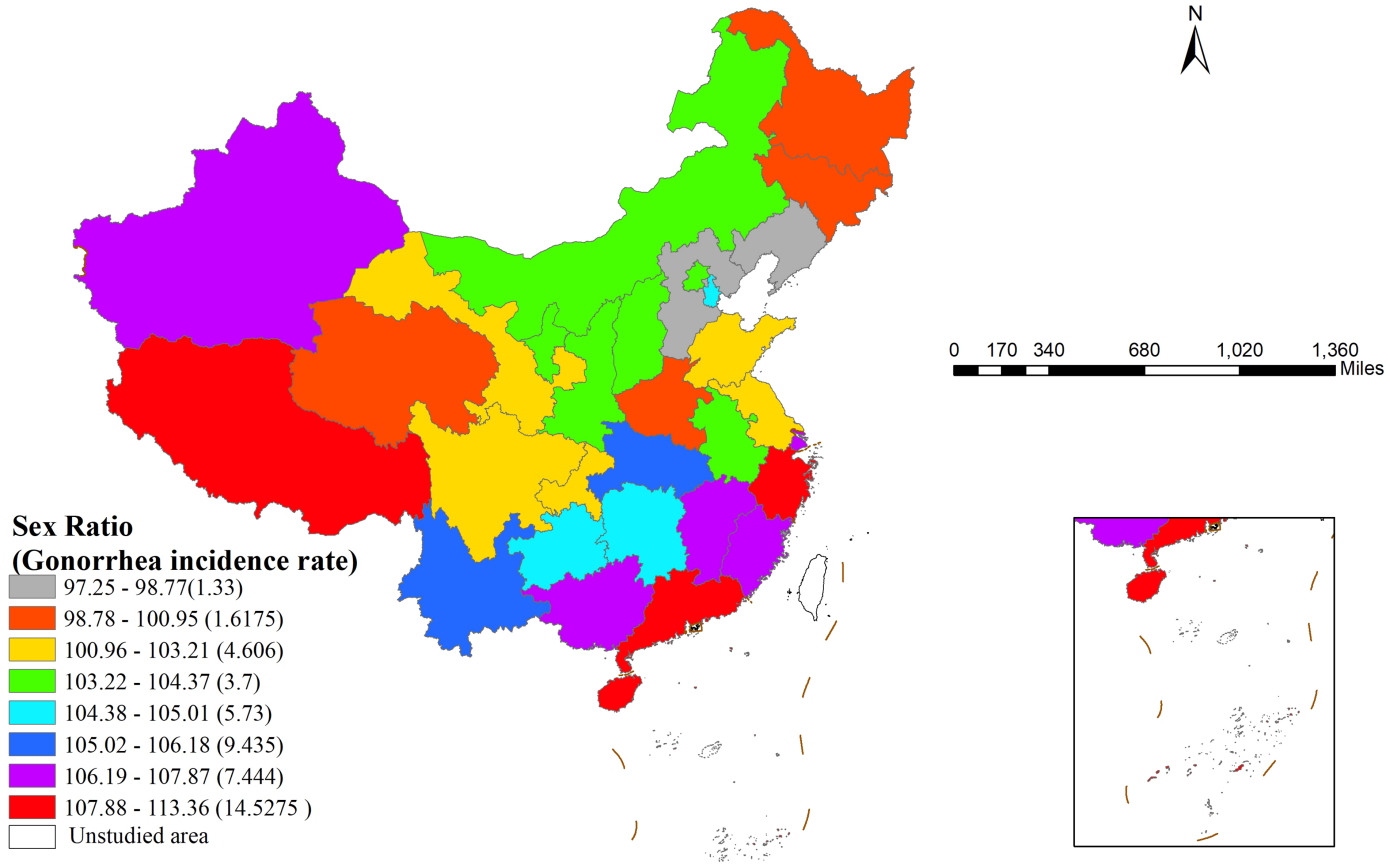
Geographic distribution of sex ratio intervals and their corresponding gonorrhea incidence rates in China.

The risk detector revealed the average gonorrhea incidence rates across different strata of each factor and analyzed whether significant differences existed between these strata. For example, the relationship between the sex ratio and gonorrhea incidence is illustrated in Figure 3. As shown in the figure, the sex ratio exhibited a clear interval effect on gonorrhea incidence: in the high sex ratio interval (107.88–113.36), the incidence rate peaked (14.5275 cases per 100,000 population), whereas in the low sex ratio interval (97.25–98.77), the incidence rate dropped to its lowest level (1.33 cases per 100,000 population).

Table 5 presents the differences in gonorrhea incidence across various sex ratio intervals using significance testing (Y/N). The study divided the sex ratio into eight gradients, with the eighth stratum (107.88–113.36) representing the highest sex ratio interval and the first stratum (97.25–98.77) representing the lowest. Statistical analysis showed that the highest sex ratio stratum (8th) exhibited significant differences in gonorrhea incidence compared to the first four low sex ratio strata (1st–4th).

**Table 5:** Statistical significance of pairwise comparisons in mean gonorrhea incidence rates across different strata of the sex ratio factor where y denotes a significant difference and n denotes no significant difference.

Stratum	1	2	3	4	5	6	7	8
1								
2	N							
3	Y	N						
4	Y	Y	N					
5	N	N	N	N				
6	N	N	N	N	N			
7	Y	Y	N	N	N	N		
8	Y	Y	Y	Y	N	N	N	

**Figure 4:**
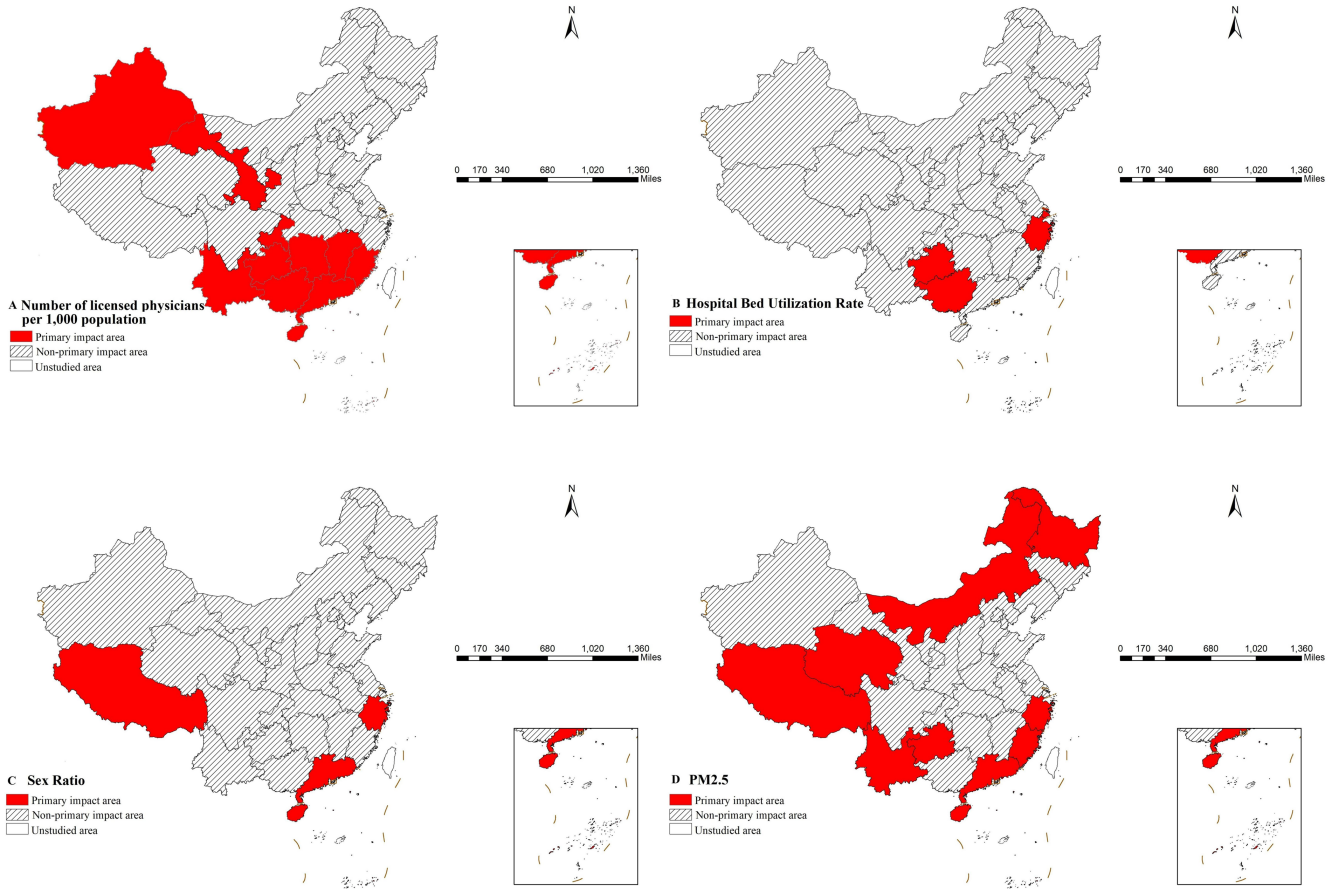
Geographic distribution of the primary impact areas for the key identified risk factors influencing gonorrhea incidence. **(A)** Number of licensed physicians per 1,000 population, **(B)** Hospital bed utilization rate, **(C)** Sex ratio, **(D)** PM_2.5_ concentration.

Further analysis demonstrated that the risk detector could quantify the quantitative relationship between influencing factors and gonorrhea incidence. As shown in Table 6, each factor had specific risk intervals corresponding to peak incidence levels (33). Epidemiologically, these high-risk intervals can be defined as the primary influence domains of each factor (34). Based on spatial analysis techniques, this study visualized these quantitative relationships in Figure 4.

**Table 6:** Identified high-risk intervals for key factors and their corresponding maximum gonorrhea incidence rates based on the risk detector analysis.

Factors	High-risk Interval	Maximum gonorrhea incidence rate (1/100,000)
Number of licensed physicians per 1,000 population	2.50–3.01	9.40
Hospital bed utilization rate(%)	65.91–78.60	12.175
Sex ratio(%)	107.88–113.36	14.5275
PM_2.5_(ug/m^3^)	8.71–24.66	9.968

#### 3.4.4 Ecological detector results

This study employed the ecological detector method to conduct pairwise comparative analysis of key factors influencing gonorrhea incidence. As shown in Table 7, statistical results indicated that only two pairs of factors exhibited statistically significant differences in PD values: sex ratio vs. physicians per 1,000 population (*p* < 0.05, marked as “Y”) and sex ratio vs. PM_2.5_ concentration (*p* < 0.05, marked as “Y”). The differences between other factor pairs were not statistically significant (marked as “N”). To further investigate the interaction effects among influencing factors and their synergistic mechanisms on gonorrhea incidence subsequent analysis will employ the interaction detector for in-depth exploration.

**Table 7:** Significance testing for differences in the power of determinism values between pairs of influential risk factors where y indicates a statistically significant difference and n indicates no significant difference.

Factors	Number of licensed physicians per 1,000 population	Hospital bed utilization rate	Sex ratio	PM_2.5_
Number of licensed physicians per 1,000 population				
Hospital bed utilization rate	N			
Sex ratio	Y	N		
PM_2.5_	N	N	Y	

#### 3.4.5 Interaction detector results

The interaction detector analysis revealed significant synergistic effects among influencing factors. As shown in Table 8, the interactive PD values for all pairwise factor combinations were substantially higher than the independent effects of single factors (PD > 0.6). Notably, the interaction between bed utilization rate and sex ratio showed the most pronounced effect (PD = 0.96). Of particular interest was the unique nonlinear enhancement characteristic observed in the interaction between number of licensed physicians per 1,000 population and PM_2.5_, while other variable combinations exhibited typical bivariate enhancement effects. This finding provides critical insights for understanding the multifactorial synergistic mechanisms underlying gonorrhea incidence.

**Table 8:** Interaction effects between pairs of factors on gonorrhea incidence rate demonstrating the magnitude of their combined influence.

Factors	Number of licensed physicians per 1,000 population	Hospital bed utilization rate	Sex ratio	PM_2.5_
Number of licensed physicians per 1,000 population	0.27			
Hospital bed utilization rate	0.61	0.50		
Sex ratio	0.88	0.96	0.62	
PM_2.5_	0.65	0.83	0.77	0.31

## 4 Discussion

The analysis reveals that gonorrhea incidence in China exhibits significant spatial clustering, with distinct high-high clusters concentrated in southern coastal provinces and low-low clusters in northern regions. Both MLR and SEM identify sex ratio, bed utilization rate, illiteracy rate, and PM_2.5_ concentration as significant factors, with SEM demonstrating superior performance. Geodetector results further quantify these relationships, showing sex ratio as the strongest individual determinant and revealing particularly powerful interactive effects, especially between bed utilization rate and sex ratio.

These patterns carry substantial implications for public health planning and intervention strategies. The introduction of the SEM significantly improved both the significance of latent factors and the model goodness-of-fit, confirming the superiority of SEM in addressing spatial issues. Compared to MLR regression, SEM effectively controls for spatial dependency bias and provides more accurate estimations and statistical inferences (26, 35). Spatial analysis revealed a significant negative spatial spillover effect in gonorrhea incidence (*λ* = −0.982, *p* < 0.001), indicating that a 1% increase in incidence in neighboring regions may lead to a 0.98% decrease in the local area. This inverse dependency may be attributed to a “siphon effect” caused by concentrated medical resources, which could render traditional joint prevention strategies less effective and thus call for dynamic monitoring and tailored resource allocation.

Notably, compared to SEM, the Geodetector effectively identified the independent influence of four key factors on gonorrhea incidence. By quantifying the driving intensity of each factor through PD-values, Geodetector circumvents the issue of multicollinearity among variables. This characteristic grants Geodetector a unique advantage in analyzing spatially heterogeneous influencing factors, particularly in handling complex spatial data while avoiding the limitations of traditional spatial regression models.

Both MLR and SEM consistently demonstrated a positive association between sex ratio and gonorrhea incidence, with factor detector analysis further identifying sex ratio as exhibiting the highest PD value. This observed relationship may be attributed to two primary factors: males are more likely to develop symptomatic infections that prompt healthcare-seeking behavior (36), and male populations (particularly men who have sex with men) generally exhibit higher prevalence of high-risk sexual behaviors (37, 38). The identification of sex ratio as the dominant driver, coupled with its specific high-risk intervals, highlights the need for targeted interventions in regions with imbalanced sex ratios, potentially focusing on male-specific health education and screening programs.

Notably, the number of physicians per 1,000 population showed no significant association in spatial regression models and yielded the lowest PD value in factor detection analysis, while bed utilization rate demonstrated relatively stronger explanatory power. This discrepancy suggests that bed utilization rate may serve as a more comprehensive indicator reflecting both healthcare facility workload and population health status (39), whereas physician density primarily represents basic healthcare coverage (40). The significant spatial dependence in gonorrhea distribution patterns, particularly evident in high-high clustering regions along the southeastern coast, likely stems from cross-regional healthcare-seeking behavior among gonorrhea patients, leading to case concentration in areas with abundant medical resources. Consequently, this phenomenon results in a decoupling effect between local physician availability and reported incidence rates. These findings carry significant implications for public health planning, suggesting that simply increasing the number of physicians may be insufficient. Instead, health authorities should prioritize enhancing the capacity and efficiency of existing healthcare facilities, as reflected by bed utilization, and establish regional referral networks to manage cross-border patient flows effectively.

This study presents the first evidence of a significant negative correlation between PM_2.5_ exposure and gonorrhea incidence. The novel finding may be mediated through two plausible mechanisms: first, as proposed in previous literature, air pollution may reduce outdoor activities and social contact opportunities, thereby potentially decreasing sexual transmission risks (41), while pollution-induced respiratory discomfort could further suppress intimate contact behaviors (42). Additionally, other pathways should be considered, such as potential confounding by regional socioeconomic characteristics or healthcare-seeking patterns that co-vary with pollution levels. Notably, industrial areas with severe PM_2.5_ pollution typically exhibit unique population mobility patterns (43) that could influence case reporting accuracy. While the observed association requires further validation, this discovery not only expands the research paradigm regarding environmental factors in sexually transmitted diseases, but more importantly highlights the necessity to consider atmospheric pollution’s potential regulatory effects on human behavioral patterns in public health research. From a policy perspective, this unexpected association underscores the complexity of disease drivers and suggests that integrated health policies, which consider environmental, social, and behavioral factors simultaneously, may be more effective than siloed approaches.

The observed inverse relationship between illiteracy rate and gonorrhea incidence may stem from data biases, confounding factors, distinctive social structures, or alternative public health interventions. This phenomenon generally reflects context-specific indirect associations that require comprehensive evaluation incorporating cultural, policy, and data quality considerations. Furthermore, all statistical models consistently demonstrated non-significant effects of urbanization rate on gonorrhea incidence distribution (*p* > 0.05), suggesting its potential pathways might be obscured by other socioeconomic factors or require more granular data for proper validation.

The interaction detector analysis revealed significant synergistic enhancement effects among influencing factors, demonstrating systematically higher PD values for all interaction terms compared to individual factors. Notably, the interaction between bed utilization rate and sex ratio exhibited the strongest synergistic effect. This phenomenon may stem from a positive feedback mechanism between gender-related biological susceptibility (higher in males) and healthcare system pressure (characterized by bed utilization rate): in regions with strained medical resources, delayed diagnosis and treatment in male patients may lead to prolonged infectious periods, thereby accelerating transmission through sexual networks (44). This finding has direct implications for public health practice. It argues against one-size-fits-all interventions and instead supports the development of integrated strategies that concurrently address demographic risks (e.g., through targeted male screening) and strengthen healthcare system capacity (e.g., by reducing diagnostic and treatment delays in high-pressure facilities), particularly in identified high-risk clusters.

This study innovatively integrates spatial regression models with Geodetector methods to systematically elucidate the spatial distribution patterns and driving mechanisms of gonorrhea incidence in China. The approach not only quantifies risk thresholds for key influencing factors but also, for the first time, reveals multifactorial synergistic effects. This coupled analytical framework of multiple models provides methodological innovation for deciphering multiscale mechanisms underlying complex public health issues.

This study has several limitations that should be acknowledged. First, at the data level, the unavailability of certain potential influencing variables may introduce omitted-variable bias into the models. Specifically, key behavioral and demographic factors such as high-risk sexual behavior frequency and migrant population size, which are known to influence gonorrhea transmission dynamics, were not included in our analysis due to data unavailability. The absence of these variables may limit the explanatory power of our current models. More importantly, a key limitation lies in the analytical scale. Our analysis relied on provincial-level data from 31 administrative units in mainland China. While this provides a broad national overview, it inevitably obscures the finer-scale spatial heterogeneity that exists at the prefectural or county levels. These factors could potentially affect the generalizability of the research findings. Therefore, future studies are advised to utilize finer-grained data (e.g., at the prefectural or county scale) to better capture local variations and enhance the model’s explanatory power.

## 5 Conclusion

In China, Gonorrhea incidence distribution exhibits significant spatial heterogeneity and autocorrelation, with the SEM demonstrating superior performance over MLR in controlling spatial dependence. The study identifies sex ratio as a core determinant of gonorrhea patterns, with male-dominated regions showing significantly higher incidence rates. Notably, bed utilization rate emerges as a stronger predictor of disease risk than physician density, underscoring the need for optimized allocation of specialized medical resources and coordinated regional prevention strategies.

A negative association was observed between PM_2.5_ exposure levels and gonorrhea incidence, suggesting environmental factors may play an underrecognized role in sexually transmitted disease transmission dynamics. The interaction effects among influencing factors significantly amplified the impact of individual factors, with the synergistic effect between bed utilization rate and sex ratio being particularly prominent. This highlights the critical importance of coordinated planning between demographic structure and healthcare resource allocation in public health policy formulation. These findings provide empirical support for developing spatially adaptive intervention frameworks that account for both sociodemographic drivers and environmental contexts in gonorrhea prevention.

## Data Availability

The original contributions presented in the study are included in the article/Supplementary material, further inquiries can be directed to the corresponding author.

## Footnotes

1
https://www.esri.com/en-us/home

2
https://geodacenter.github.io/

3
http://geodetector.cn/Download.html

4
https://www.tianditu.gov.cn/
